# Bispecific antibodies revolutionizing breast cancer treatment: a comprehensive overview

**DOI:** 10.3389/fimmu.2023.1266450

**Published:** 2023-12-04

**Authors:** Huan-Rong Lan, Min Chen, Shi-Ya Yao, Jun-Xia Chen, Ke-Tao Jin

**Affiliations:** ^1^ Department of Surgical Oncology, Hangzhou Cancer Hospital, Hangzhou, Zhejiang, China; ^2^ Department of Colorectal Surgery, Sir Run Run Shaw Hospital, Zhejiang University School of Medicine, Hangzhou, Zhejiang, China; ^3^ Department of Colorectal Surgery, Affiliated Jinhua Hospital, Zhejiang University School of Medicine, Jinhua, Zhejiang, China; ^4^ Department of Gynecology, Shaoxing People’s Hospital, Shaoxing, Zhejiang, China

**Keywords:** breast cancer, bispecific antibodies, targeted therapy, immunotherapy, tumor specificity

## Abstract

Breast cancer (BCa) is known as a complex and prevalent disease requiring the development of novel anticancer therapeutic approaches. Bispecific antibodies (BsAbs) have emerged as a favorable strategy for BCa treatment due to their unique ability to target two different antigens simultaneously. By targeting tumor-associated antigens (TAAs) on cancer cells, engaging immune effector cells, or blocking critical signaling pathways, BsAbs offer enhanced tumor specificity and immune system involvement, improving anti-cancer activity. Preclinical and clinical studies have demonstrated the potential of BsAbs in BCa. For example, BsAbs targeting human epidermal growth factor receptor 2 (HER2) have shown the ability to redirect immune cells to HER2-positive BCa cells, resulting in effective tumor cell killing. Moreover, targeting the PD-1/PD-L1 pathway by BsAbs has demonstrated promising outcomes in overcoming immunosuppression and enhancing immune-mediated tumor clearance. Combining BsAbs with existing therapeutic approaches, such as chemotherapy, targeted therapies, or immune checkpoint inhibitors (ICIs), has also revealed synergistic effects in preclinical models and early clinical trials, emphasizing the usefulness and potential of BsAbs in BCa treatment. This review summarizes the latest evidence about BsAbs in treating BCa and the challenges and opportunities of their use in BCa.

## 1 Introduction

Breast cancer (BCa) remains a significant global health concern, demanding the development of innovative and effective therapeutic strategies (1). Bispecific antibodies (BsAbs) have emerged as a promising approach to treating BCa, offering unique capabilities for targeted therapy and immunomodulation (2). BsAbs are engineered molecules designed to bind two antigens simultaneously (3). This feature targets tumor-associated antigens (TAAs) on BCa cells while engaging immune effector cells or blocking critical signaling pathways. By harnessing this dual targeting ability, BsAbs can enhance tumor specificity and induce robust immune responses against BCa cells (3).

Several preclinical and clinical studies have demonstrated the potential of BsAbs in BCa treatment. For instance, BsAbs targeting HER2 have shown the ability to redirect immune cells to HER2-positive BCa cells, resulting in a potent tumor cell-killing (4, 5). Additionally, BsAbs targeting immune checkpoint molecules, such as PD-1 or PD-L1, have shown promising results in overcoming immunosuppression and enhancing immune-mediated tumor clearance (6, 7). Combining BsAbs with conventional therapies, including chemotherapy or targeted agents, has also shown synergistic effects in preclinical models and early clinical trials (8, 9). These combination strategies hold great potential for improving treatment outcomes in BCa patients. Therefore, BsAbs represent a promising therapeutic approach in BCa treatment (10). Their ability to simultaneously target tumor cells and engage the immune system offers the potential for enhanced tumor specificity and improved anti-cancer activity (11).

Further research and clinical investigations are warranted to optimize BsAb design, dosing, and combination strategies. By harnessing the potential of BsAbs, we may witness significant advancements in managing BCa and ultimately improve patient outcomes (12, 13). This review aims to explore the potential of BsAbs in treating BCa by examining its mechanisms of action, preclinical and clinical evidence, and future prospects.

## 2 Breast cancer

BCa is considered a complex and heterogeneous breast tissue disease (14). It is one of the most frequent malignancies in women but can also occur in men, although it is less common (15). Understanding the different subtypes of BCa is essential for tailoring treatment approaches to individual patients (16). This information can help in accurate diagnosis, treatment planning, and patient prognosis (17).

### 2.1 Molecular subtypes of breast cancer

Human epidermal growth factor receptor 2 (HER2)-positive BCa is characterized by the overexpression of the HER2 protein (18). These tumors grow more rapidly and have a poorer prognosis (19). However, targeted therapies such as trastuzumab (Herceptin) have significantly improved outcomes for patients with HER2-positive BCa (20). Triple-negative breast cancer (TNBC) is another BCa subtype characterized by the absence of and HER2 expression, estrogen receptor (ER), and progesterone receptor (PR) (21). This subtype is more aggressive and has fewer targeted treatment options available, accounting for 10-15% of BCas (22). Luminal A and Luminal B BCas are subtypes characterized by the presence of hormone receptors (ER and/or PR) (23). Luminal A tumors have a low proliferative rate and tend to have a better prognosis (24). In contrast, Luminal B tumors have a higher proliferative rate and are associated with a slightly worse prognosis than Luminal A (24).

### 2.2 Histopathologic classifications

One subtype of BCa is ductal carcinoma *in situ* (DCIS), originating in the milk ducts (25). It is considered non-invasive, as the abnormal cells are confined to the ducts and have not spread to nearby tissues (26). However, if left untreated, DCIS can progress to invasive BCa. The most common subtype of invasive BCa is invasive ductal carcinoma (IDC), accounting for approximately 70-80% of cases (27). IDC begins in the milk ducts and invades the surrounding breast tissue. This subtype can be further categorized based on hormone receptor status and HER2 expression (28). Invasive lobular carcinoma (ILC) is another subtype that starts in the milk-producing glands (lobules) and can spread to other parts of the breast and beyond (29). It accounts for approximately 10-15% of invasive BCas and has distinct characteristics and patterns of growth compared to IDC (30). Inflammatory BCa (IBC) is a rare and aggressive subtype (31). It accounts for approximately 1-5% of BCa cases (32). Unlike other subtypes, IBC presents symptoms such as redness, swelling, and warmth in the breast, giving it a distinct appearance (33). Immediate and aggressive treatment is required for IBC.

Advances in research and molecular profiling have helped identify these subtypes and develop targeted treatments, leading to improved outcomes for patients with BCa (34–36). Each subtype has unique characteristics and responses to specific therapies.

## 3 Bispecific antibodies: structures and mechanisms of action

BsAbs are a class of engineered antibodies that can simultaneously bind to two targets, often two distinct antigens or receptors (37). They are designed to redirect immune cells or deliver therapeutic payloads to specific cells or tissues, offering a versatile approach to treating various human disorders. BsAbs have gained significant attention and promise in medicine due to their unique targeting abilities and potential applications in treating multiple diseases (38). The design of BsAbs involves combining specific binding domains from two different mAbs into a single molecule. This allows them to interact with two different targets simultaneously, facilitating various therapeutic strategies (39).

### 3.1 Different forms of bispecific antibodies

**Figure 1:**
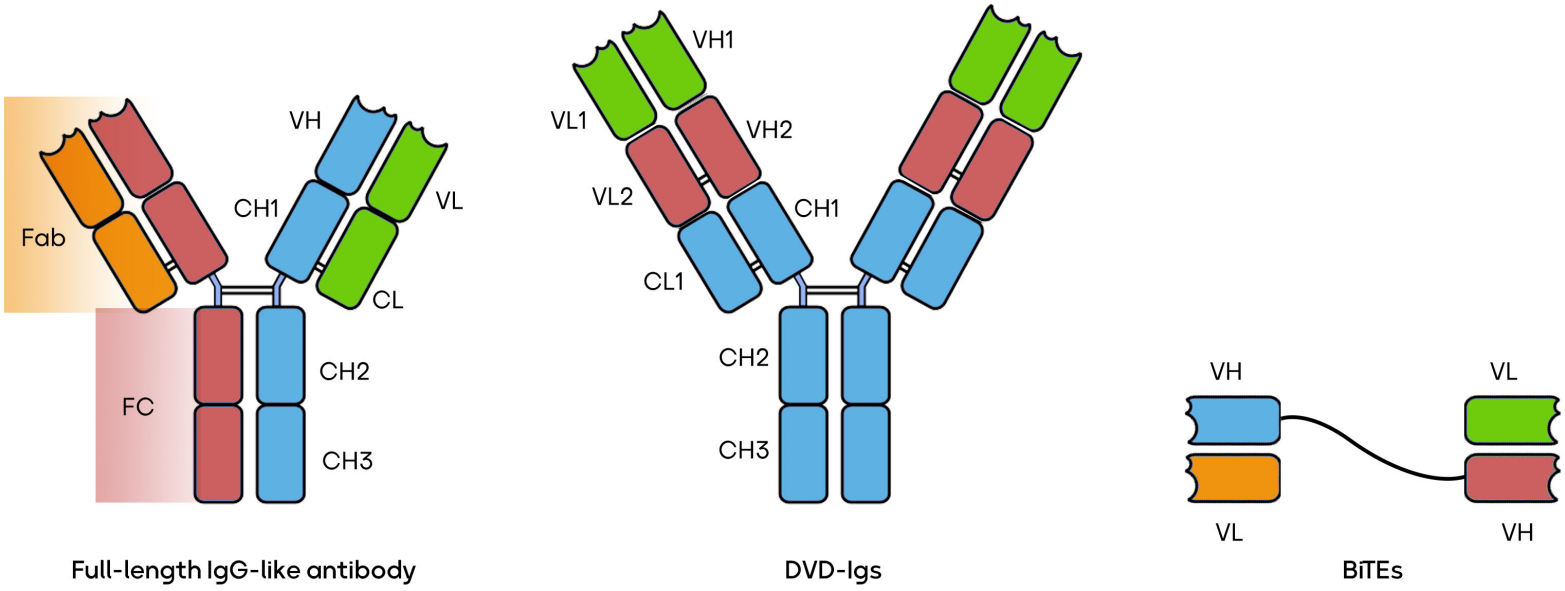
Three types of BsAbs.

The world of BsAbs is a burgeoning field offering a versatile array of therapeutic possibilities. BsAbs come in various constructions, each tailored to address specific medical needs. Some, like the IgG-like BsAbs, closely mimic natural antibodies, exemplified by catumaxomab’s application in ovarian cancer (40). Others, such as CrossMabs, connect different antibody fragments to target multiple antigens simultaneously, as seen with CEA-TCB in colorectal cancer (41). T-cell engagers like blinatumomab recruit and activate T-cells to combat leukemia (5). Dual-variable-domain (DVD) antibodies incorporate two antigen-binding domains within a single heavy chain, with RG6110 being a candidate for HER2-positive tumors (42). Moving beyond, tri-specific antibodies, exemplified by AFM13 in Hodgkin lymphoma, target three antigens for even greater specificity (43). BsAbs can be conjugated to cytotoxic drugs, like ABBV-838 for solid tumors (44), or combined with checkpoint inhibitors, e.g., Epcoritamab for B-cell malignancies (45). Immune stimulators, such as Cibisatamab with a 4-1BB agonist for colorectal cancer (46), and applications beyond cancer, like Emicizumab for hemophilia A (47), further illustrate the diversity and promise of BsAbs. These innovations can potentially revolutionize targeted therapies across a spectrum of diseases, marking a pivotal era in biotechnology. Several forms of BsAbs, including full-length IgG-like antibodies, bispecific T-cell engagers (BiTEs), and dual-variable domain immunoglobulins (DVD-Igs) are shown in **Figure 1** (39, 48, 49).

### 3.2 Bispecific antibodies mechanism of action

The primary application of BsAbs lies in cancer immunotherapy (50). BsAbs can enhance the anti-tumor immune response by targeting cancer cells and engaging the immune system (44). Here, it has been discussed how BsAbs can induce anti-tumor immune responses and affect tumor cells. BsAbs are designed to recognize two distinct antigens: one on the surface of tumor cells and another on immune cells. This dual targeting allows BsAbs to bridge the gap between tumor and immune cells, bringing them into close proximity (11). The antigen recognized on the tumor cell surface by one arm of the BsAb is often a specific marker associated with the tumor. This binding can trigger various mechanisms for tumor cell killing. In this context, it has been revealed that BsAbs can induce apoptosis in tumor cells by cross-linking them with immune cells, such as cytotoxic T cells or natural killer (NK) cells (51). This activates the immune cells to release cytotoxic molecules like perforin and granzymes, which damage the tumor cell membrane and lead to cell death. The binding of the BsAb to the tumor cell can also recruit immune cells, particularly NK cells, to the tumor site (52). These NK cells can recognize the Fc portion of the BsAb and induce antibody-dependent cell cytotoxicity (ADCC), leading to the lysis of the tumor cell (52). Some BsAbs are engineered to activate the complement system, a part of the immune system that can cause cell lysis. When the BsAb binds to the tumor cell and activates complement, it forms a membrane attack complex (MAC) that punches holes in the tumor cell membrane, resulting in cell death (53, 54). By binding to an antigen on immune cells, the other arm of the BsAb can activate these immune cells. For example, it can engage with T cells and provide a co-stimulatory signal that enhances their activation and proliferation. This helps boost the immune response against the tumor. BsAbs can also influence the TME. They can help reduce immunosuppressive factors and promote an inflammatory response within the tumor, making it more susceptible to immune attack (55). Moreover, BsAbs can facilitate the uptake and presentation of tumor antigens by antigen-presenting cells (APCs), such as dendritic cells (DCs). This can lead to a more robust adaptive immune system activation, including T-cell responses (56).

One prominent example is the approval of Blinatumomab, a BiTE antibody, for treating relapsed or refractory B-cell acute lymphoblastic leukemia (57). Blinatumomab binds to CD19 on cancer cells and CD3 on T cells, enabling T cells to recognize and eliminate malignant B cells (57). This approach has shown remarkable efficacy in clinical trials, improving patient outcomes (58). Specific targeting therapy using lymphokine-activated killer (LAK) cells treated with BsAbs appeared to be a promising and effective form of adoptive immunotherapy for malignant glioma (59). In a phase I clinical trial, four ovarian cancer patients were treated with autologous lymphocytes coated with a bispecific F(ab’)2 antibody. Fortunately, no serious side effects were reported. However, it was noted that the patients developed human anti-murine antibodies, primarily targeting the idiotype of monoclonal antibody (MOv18) (60). This finding suggests an immune response against the murine components of the BsAb used in the treatment. Monitoring and managing immune responses to therapeutic antibodies is essential in developing such treatments to ensure their safety and efficacy. Further research and clinical trials may address these immune responses to improve the treatment’s outcomes.

In addition to cancer therapy, BsAbs have shown potential in various other human disorders. For example, in autoimmune diseases such as rheumatoid arthritis, BsAbs can be engineered to simultaneously bind to an antigen expressed on the surface of auto-reactive B cells and CD3 on T cells, facilitating the depletion of these pathogenic B cells (61). This approach helps restore immune balance and reduce inflammation.

Furthermore, BsAbs hold promise in infectious disease treatment (62). They can be designed to target viral antigens and recruit immune cells, such as NK cells or macrophages, to eliminate infected cells via NK cell-mediated ADCC (62, 63). This approach has been explored for HIV, hepatitis B and C, and other viral infections (62, 64, 65). Neurology is another area where bi-specific antibodies have shown potential (66–68). A study reported a hypothesis that using anti-CD3 activated T cells (ATCs) armed with a chemically heteroconjugated anti-CD3 × polyclonal anti-CMV BsAb (CMVBi) could effectively target and eradicate CMV-infected cells (69). Even at low arming doses of CMVBi, the researchers found that specific cytotoxicity (SC) against CMV-infected target cells was significantly enhanced compared to unarmed ATCs, especially at various effector-to-target ratios (E: T). Armed ATCs demonstrated substantial killing of CMV-infected targets while sparing uninfected cells. Additionally, co-cultures of CMVBi-armed ATCs with CMV-infected targets triggered the release of cytokines and chemokines from the armed ATCs. This strategy represents a potential non-major histocompatibility complex restricted approach to prevent or treat CMV-related infections following organ or allogeneic stem cell transplantation (69).

BsAbs can be developed to target specific proteins involved in neurodegenerative disorders like Alzheimer’s disease or Parkinson’s disease (70, 71). By binding to the pathological proteins and engaging immune cells or facilitating clearance mechanisms, BsAbs can potentially halt disease progression or reduce the accumulation of toxic aggregates (72, 73).

The efficacy of single-chain variable fragment (scFv) versus bivalent targeting for T cell-mediated killing of TAAs can vary depending on several factors, including the specific target antigen, the construct’s design, and the immune response context (74). ScFv-based constructs consist of a single chain of variable regions of an antibody, while bivalent constructs typically include a dimeric or multimeric format with dual antigen-binding sites (75, 76). The choice between these constructs often depends on the antigen density on the surface of target cells and the need for avidity. In cases where the TAA is highly expressed, bivalent targeting can enhance T cell activation and cytotoxicity due to increased antigen crosslinking, potentially leading to a more potent killing (77). However, for targets with lower antigen density or minimizing off-target effects is crucial, scFv-based constructs may be preferred as they provide specificity while reducing the risk of off-target binding (78).

An investigation has revealed that the IgG-[L]-scFv BsAb platform significantly improves the ability of T cells armed with BsAbs to combat tumors. Compared to the separate administration of BsAbs and T cells, using BsAb-armed T cells, known as EATs, led to reduced tumor necrosis factor-alpha (TNF-α) release, quicker tumor infiltration, and strong antitumor responses. The effectiveness of EAT therapy *in vivo* was influenced by factors like the dose of BsAbs used for arming, the quantity of EAT cells per injection, the total number of EAT doses, and the treatment schedule’s intensity. Importantly, the antitumor potency of EATs remained intact even after cryopreservation and EATs employing γδ T cells were demonstrated to be both safe and as effective as αβ T cell-based EATs. This research highlights the potential of EATs as a promising avenue for cancer treatment (79).

The development and approval of BsAbs have been relatively slow despite over 25 years of engineering and clinical trials due to several challenges, including complex design, manufacturing, and safety concerns. BsAbs require precise engineering to ensure proper targeting and minimal off-target effects, making their development more time-consuming and resource-intensive. Additionally, manufacturing BsAbs can be challenging, as they often involve the production of two different binding domains within a single molecule. Safety concerns, such as cytokine release syndrome (CRS) and on-target/off-tumor toxicities, have also slowed their progress through clinical trials (80).

Collectively, BsAbs represent a powerful therapeutic approach with diverse applications in human disorders. Their ability to simultaneously target multiple antigens or receptors provides enhanced specificity and efficacy compared to traditional mAbs. As research and development in this field continue to advance, BsAbs hold great promise for improving the treatment outcomes of various diseases and transforming the landscape of medicine.

## 4 Known bispecific antibodies in breast cancer treatment

**Figure 2:**
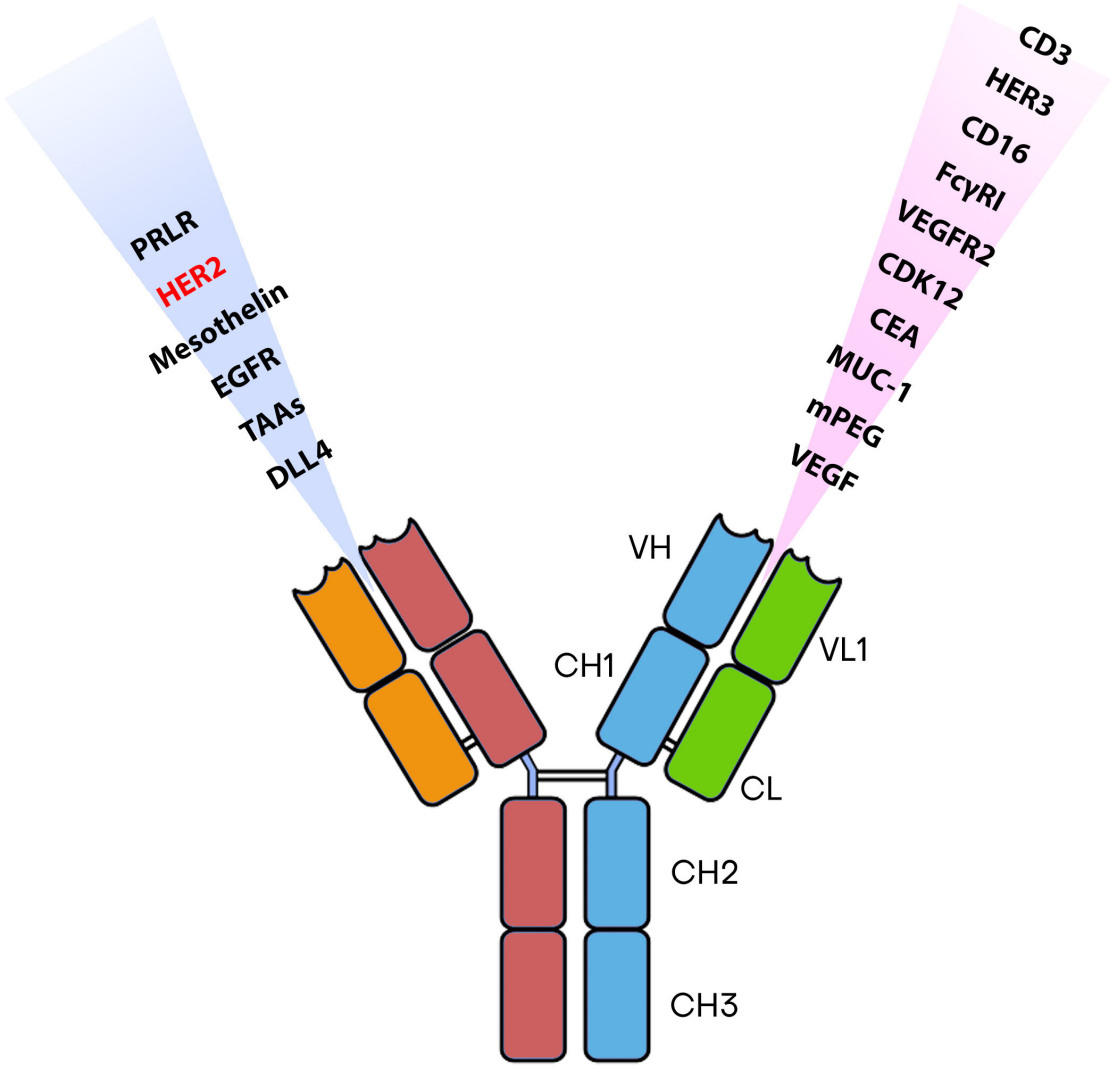
The most commonly identified antigens for designing BsAbs.

Numerous BsAbs are currently undergoing development and possess diverse designs that hold significance concerning BCa. BsAbs dedicated to BCa entails agents that effectively direct immune recognition toward cancer cells, aim at specific cancer antigens, and target the microenvironment associated with the disease (**Figure 2**). These BsAbs are being meticulously crafted for their potential utilization as antibody-drug conjugates and as molecular cues to guide engineered T-cells toward their intended targets (81) (**Table 1**). MM-111’s ability to simultaneously bind to both HER2 and HER3 receptors provides a means to disrupt downstream signaling pathways, while ertumaxomab enhances the interaction between immune effector cells and tumor cells (99). These BsAbs hold promise as they target multiple pathways involved in HER2-positive cancers, potentially overcoming resistance. Furthermore, using activated T cells armed with anti-HER2 BsAbs (HER2Bi-aATC) presents another avenue for treatment. This approach leverages the power of the immune system to target HER2-expressing cancer cells directly (10).

**Table 1:** The most important BsAbs in treating BCa.

BsAbs	BsAbs Targets	Details of study	Outcomes	Ref
**TP_L_**	HER2 epitops BsAb Sources: trastuzumab and pertuzumab	*In vitro* BT-474 SK-BR-3 HCC-1954 MDA-MB-231 MDA-MB-468 and MCF-7 *In vivo* female BALB/c mice	•Superior blocking action against HER2 heterodimerization compared to the combination of trastuzumab and pertuzumab •Effectively inhibits HER2 signaling in trastuzumab-resistant BCa cell lines •Outperforms trastuzumab plus pertuzumab in inhibiting the growth of trastuzumab-resistant BCa cell lines •Eradicates established trastuzumab-resistant tumors in mice	(82)
**p95HER2-TCB**	P95HER2 and CD3ϵ	*In vitro* MCF7 MCF10A Jurkat cells *In vivo* Humanized xenograft models	•Potent anti-tumor effects on primary BCas and brain lesions that express p95HER2 •Unlike TCBs targeting HER2 the p95HER2-TCB had no impact on nontransformed cells that do not overexpress HER2	(83)
**Four types of BsAbs**	HER2 and CD3 IgG-based bsAbs	*In vitro* SKBR3 Her2 3 +; MDA MB453 Her2 2 +; MDA MB231 Her2 1 +; MDA MB468 Her2 0 *In vivo* xenograft NGS mice model	•Different valencies of the BsAbs did not significantly impact their effectiveness in fighting tumors •Fc domain enhanced the BsAbs’ ability to induce cytotoxic activity against the cancer cells •The Fc domain also triggered T-cell activation in a manner unrelated to the presence of the target antigen •The BsAbs efficiently redirected T cells to effectively eliminate all cancer cells expressing HER2 including those with low levels of HER2 expression	(84)
**BiMAbs**	HER2/EGFR/CEA/EpCAM and αCD3/αCD28 IgG1-Fc based format	*In vitro* MCF-7 HT-1080/FAP	•Effectively activated T cells and induced cytotoxicity only in the presence of tumor cells •Combination treatment with αTAA–αCD3 BiMAb and co-stimulatory αTAA–αCD28 or αTAA–TNFL fusion proteins significantly enhanced T cell activation proliferation activation marker expression cytokine secretion and tumor cytotoxicity	(85)
**HER2-BsAb**	HER2 and CD3	*In vitro* HCC1954 *In vivo* BALB-Rag2^−/−^IL-2R-γc-KO (DKO) mice	•Promoted of T-cell infiltration and suppression of tumor growth mainly when used in conjunction with human PBMC or ATC	(86)
**BAb**	CEA and HER2 Murine IgG1 subclass	*In vitro* SKOv3-CEA-1B9 *In vivo* Double-positive tumour-bearing nude mice	•Enhanced tumor localization compared to single-specificity antibodies	(87)
**DF3xH22**	MUC-1 and HER2	*In vitro* R75-1 MCF-7 BT-20 T-47D SKBR-3	•Mediated the phagocytosis of MUC-1-expressing target cells •Inducing ADCP	(88)
**BsAb; mPEG × HER2**	mPEG and HER2 Anti-HER2 scFv and anti-DNS scFv	*In vitro* MCF7/HER2 (HER2^high^) and MCF7/neo1 (HER2^low^) *In vivo* BALB/c nude mice	•One-step formulation of PLD using mPEG × HER2 enhanced tumor specificity increased drug internalization and improve the anticancer activity of PLD against HER2-overexpressing and doxorubicin-resistant BCa	(89)
**TC-BsAb**	EGFR and HER2	*In vitro* BT-474 and SK-BR-3 *In vivo* female BALB/c nude mice	•Demonstrated significantly greater potency in inhibiting the growth of BCa cell lines compared to trastuzumab cetuximab and the combination of trastuzumab plus cetuximab	(90)
**Anti-EGFR/VEGFR2 BsAb**	EGFR and VEGFR2 Cetuximab IgG linked to the scFv of ramucirumab via a glycine linker	*In vitro* MDA-MB-231 BT-20 MDA-MB-468 BT549 and HS578 T *In vivo* female athymic nude mice	•Inhibited EGFR and VEGFR2 in TNBC cells disrupting the autocrine mechanism •Inhibited ligand-induced activation of VEGFR2 and blocked the paracrine pathway mediated by VEGF secreted from TNBC cells in endothelial cells	(91)
**HB-32**	DLL4 and VEGF Derived from Bevacizumab and H3L2 was use as the parental mAb The anti-DLL4 antibody (H3L2) was generated using the hybridoma technique and humanized transformation	*In vitro* MDA-MB-231 cells *In vivo* BALB/c nude mice	•Effectively inhibited the proliferation migration and tube formation of HUVEC which are involved in angiogenesis •HB-32 inhibited the proliferation of BCa cells and induces tumor cell apoptosis more effectively than treatment with an anti-VEGF antibody or an anti-DLL4 antibody alone	(92)
**HER2xPRLR bispecific ADC**	HER2 and PRLR A fully human mAb to human PRLR and “in-house trastuzumab”	*In vitro* HEK293 cells	•Significantly enhanced the degradation of HER2 and the cell-killing activity of a noncompeting HER2 ADC—in BCa cells that coexpressed HER2 and PRLR	(93)
**PRLR-DbsAb**	PRLR and CD3	*In vitro* MDA-MB-231 MCF-7 and SKBR-3 cells *In vivo* Female NOD/SCID mice	•Activated T cells and stimulated the release of antitumor cytokines •Showed significant inhibition of tumor growth and increased survival compared to traditional mAb treatment	(94)
**MDX-21**	HER2 and FcγRI (CD64)	*In vitro* SK-BR-3 BT-20 T-47D	•Induce phagocytosis and cytolysis of BCa cells by human MDMs •Induced ADCP and ADCC •Combining MDX-H210 and G-CSF did not demonstrate significant therapeutic efficacy regarding clinical responses •Isolated neutrophils from patients undergoing G-CSF treatment displayed high cytotoxicity in the presence of MDX-210	(95)
**MesobsFab**	Mesothelin and FcγRIII (CD16)	*In vitro* BT-474 HCC1806 SK-BR-3 and MDA-MB-231 *In vivo* Humanized xenograft models	•Facilitated the recruitment and infiltration of NK cells into tumor spheroids •Induced ADCC •Elicited dose-dependent cell-mediated cytotoxicity against mesothelin-positive tumor cells •Induced cytokine secretion •Reduced cell invasiveness	(96)
**HER2bsFab**	HER2 and FcγRIII (CD16) Fab-like BsAb	*In vitro* SK-OV-3 SK-BR-3 BT-474 MCF-7	•Effectively inhibited the growth of HER2-high tumors by recruiting resident effector cells expressing mouse FcγRIII and IV •Showed superior inhibition of HER2-low tumor growth compared to trastuzumab	(97)
**BsAb**	HER2 and FcγRIII (CD16) A trivalent anti-erbB2/anti-CD16 BsAb	*In vitro* SKBR3 cells	•Activated NK cells to enhance anti-tumor immune responses	(98)

### 4.1 Preclinical studies

To assess safety and efficacy, a study combines MM-111 with trastuzumab, a standard HER2-targeted therapy. The research includes a dose-escalation phase and an expansion cohort, aiming to identify the right treatment dosage. Preliminary results indicate that the ongoing study intends to improve treatment options for HER-2-positive advanced BCa patients (100).

HER2-targeted immunotherapy has revolutionized the treatment of HER2-positive BCa, offering multiple strategies to combat the disease (101, 102). Monoclonal antibodies (mAbs) like trastuzumab have long been the standard of care, effectively targeting HER2 overexpression (103). The combination of trastuzumab, pertuzumab, and paclitaxel has shown promising results as a frontline therapy for advanced HER2-positive BCa (104). However, resistance to anti-HER2 antibodies remains challenging, necessitating the development of alternative approaches (105). Researchers have created a bispecific anti-HER2 antibody, TP_L_, to address this issue by combining trastuzumab and pertuzumab.

Pertuzumab is an additional humanized antibody with a different target site on HER2 than trastuzumab. This novel antibody, TP_L_, preserves the binding characteristics of both of its parent antibodies and exhibits pharmacokinetic properties similar to conventional immunoglobulin G molecules. TP_L_ demonstrates superior capabilities in blocking HER2 heterodimerization compared to the combination of pertuzumab and trastuzumab. This heightened performance may be due to steric hindrance or the induction of a conformational change in the HER2 protein. Importantly, TP_L_ proves effective in inhibiting HER2 signaling even in BCa cell lines that have developed resistance to trastuzumab. In both laboratory and animal experiments, TP_L_ surpasses trastuzumab and pertuzumab combined in suppressing the growth of these trastuzumab-resistant BCa cell lines. Notably, TP_L_ treatment successfully eliminates well-established trastuzumab-resistant tumors in mice. These findings strongly suggest that trastuzumab-resistant breast tumors rely heavily on HER2 signaling. They also indicate that a comprehensive blockade of HER2 heterodimerization could be a viable therapeutic approach. TP_L_’s unique potential to overcome trastuzumab resistance underscores its promise as an attractive treatment option in clinical settings. Further exploration and evaluation of TP_L_’s efficacy are warranted for its consideration as a valuable therapeutic strategy (82). Other potential solutions are MM-111 and ertumaxomab, offering distinct mechanisms of action (10).

T cell bispecific antibodies (TCBs) are engineered molecules that can bind to T cell receptor (TCR) components and TAAs, such as HER2 or tumor-specific antigens (TSAs) (106). However, TCBs targeting HER2 have been associated with severe toxicities, possibly due to HER2 expression in normal epithelial cells (107). Researchers investigated an alternative approach by targeting p95HER2, a carboxyl-terminal fragment of HER2 expressed in about 40% of HER2-positive tumors (83). They demonstrated that p95HER2 was not expressed in normal tissues, as confirmed by specific antibody analysis. The researchers successfully engineered a p95HER2-TCB, and their study demonstrated its remarkable effectiveness in combating primary BCas and brain lesions that exhibit p95HER2 expression. What’s particularly noteworthy is that, in contrast to TCBs directed at HER2, the p95HER2-TCB did not affect normal, non-transformed cells that do not exhibit HER2 overexpression (83). These findings suggest that targeting p95HER2 with TCBs could offer a safe and effective treatment strategy for a subgroup of HER2-positive tumors by selectively targeting a TSA. The findings pave the way for further research and potential clinical development of p95HER2-TCB as a targeted treatment for HER2-positive BCas expressing p95HER2.

In another investigation, a research team developed four BsAbs by combining anti-HER2 antibodies with anti-CD3 antibodies (84). These BsAbs were created using a genetically encoded noncanonical amino acid. The variations included different valencies and the presence or absence of an Fc domain. The study investigated how these variations influenced the BsAbs’ ability to target HER2-expressing cancer cells. The results showed that the different valencies of the BsAbs did not significantly impact their effectiveness in fighting tumors. However, the Fc domain enhanced the BsAbs’ ability to induce cytotoxic activity against the cancer cells. Unfortunately, the Fc domain also triggered T-cell activation in a manner unrelated to the presence of the target antigen. The study demonstrated that the BsAbs efficiently redirected T cells to eliminate all cancer cells expressing HER2, including those with low levels of HER2 expression. This was observed in laboratory experiments conducted *in vitro* and animal models (rodent xenografts) (84). This study offers valuable insights into the structural characteristics of BsAbs that impact their functionality. Additionally, it underscores the promising potential of BsAbs as a therapeutic choice for BCa patients, particularly those with low or varied HER2 expression. By proficiently targeting cancer cells that express HER2, even those with minimal HER2 levels, BsAbs present a promising avenue for enhancing BCa treatment.

A study aimed to improve the efficacy of T cell-recruiting BsAb (BiMAb) for solid TAAs in carcinomas has been challenging compared to hematologic malignancies (85). The researchers put forward a hypothesis that the combination of co-stimulatory Bispecific Monoclonal Antibodies (BiMAb) with αTAA–αCD3 BiMAb could bolster T cell activation and their ability to multiply, thus improving the targeting of tumor antigens that are expressed weakly or heterogeneously. Various combinations of αTAA–αCD3 and αTAA–αCD28 BiMAb in a tetravalent IgG1-Fc format were examined, targeting multiple BCa antigens like HER2, epithelial cell adhesion molecule (EpCAM), carcinoembryonic antigen (CEA), and epidermal growth factor receptor (EGFR). Additionally, they explored bifunctional fusion proteins of αTAA–tumor necrosis factor ligand (TNFL) superfamily members, including 4-1BBL, OX40L, CD70, and TL1A. To evaluate the functionality of these BiMAbs, the researchers conducted tests using co-cultures of tumor cell lines and purified T cells in monolayer and tumor spheroid models. The results revealed that αTAA–αCD3 BiMAb effectively activated T cells and induced cytotoxicity only in the presence of tumor cells, signifying a strict reliance on cross-linking. Furthermore, the combination treatment of αTAA–αCD3 BiMAb with co-stimulatory αTAA–αCD28 or αTAA–TNFL fusion proteins led to a significant enhancement in T cell activation, proliferation, activation marker expression, cytokine secretion, and their ability to target and destroy tumor cells (85).

Moreover, co-stimulation of BiMAb decreased the minimum needed dose for T-cell activation. The co-stimulation is able to inhibit immune-suppressive effects of interleukin (IL)-10 and tumor growth factor (TGF)-β on T cell activation and the formation of memory cells (108). Furthermore, using immune checkpoint inhibitors (ICIs) intensified the co-stimulation facilitated by BiMAb. This effective co-stimulation could be achieved by targeting a secondary BCa antigen or fibroblast activation protein (FAP) expressed on another type of target cell (109). In tumor spheroids derived from pleural effusions of BCa patients, the presence of co-stimulatory BiMAb proved to be crucial for activating tumor-infiltrating lymphocytes (TILs) and eliciting cytotoxic anti-tumor responses against BCa cells. In a broader context, the study showcased that co-stimulation significantly enhanced the ability of T cell-activating BiMAb to eliminate tumors while still relying on the recognition of TAAs. This approach has the potential to offer a more localized activation of the immune system with heightened effectiveness and reduced peripheral side effects, presenting promising prospects for enhancing immunotherapy in the treatment of solid tumors (85).

On the other hand, some studies have warned about targeting CD28 with mAbs and its fatal toxicities (110, 111). A phase I clinical trial of TGN1412, a superagonist anti-CD28 mAb, revealed severe and unexpected toxicities in healthy volunteers, highlighting the need for extreme caution when conducting trials with such agents. The rapid onset of a systemic inflammatory response, including CRS, organ failure, and a dramatic depletion of immune cells, underscored the potential dangers of novel immunomodulatory therapies. This study serves as a stark warning about the importance of rigorous preclinical evaluation and the careful design of early-phase clinical trials, emphasizing the necessity of close monitoring and promptly addressing adverse events to ensure the safety of participants. These findings indicated the imperative for thoroughly understanding and mitigating potential toxicities before advancing such therapies into human trials (112).

In this regard, a novel HER2/CD3 BsAb platform called HER2-BsAb also was designed (86). HER2-BsAb preserves the antiproliferative effects of trastuzumab, an established HER2-targeted therapy, while recruiting and activating non-specific circulating T-cells. This recruitment and activation of T-cells promote tumor infiltration and eradicate HER2-positive tumors, even those resistant to standard HER2-targeted therapies (113). In *in vitro* studies, it has been established that HER2-BsAb could have cytotoxicity against tumors. The effectiveness, measured by EC_50_ (half-maximal effective concentration), is directly related to the level of HER2 expression on the surface of various human tumor cell lines. This correlation holds regardless of the lineage or type of the tumor, emphasizing the versatility of HER2-BsAb. Crucially, the cytotoxic effects mediated by HER2-BsAb appear to be relatively resistant to PD-1/PD-L1 inhibition. This suggests that HER2-BsAb may remain effective even with ICIs. Furthermore, HER2-BsAb has demonstrated a remarkable ability to promote the infiltration of T-cells and suppress tumor growth, especially when combined with human peripheral blood mononuclear cells (PBMCs) or activated T-cells. The compelling antitumor properties observed in both *in vivo* and *in vitro* settings provide strong support for advancing the clinical development of HER2-BsAb as a potential cancer immunotherapeutic. By leveraging the unique capabilities of BsAbto engage T-cells and target HER2-positive tumors, HER2-BsAb holds potential as a valuable addition to the treatment arsenal for HER2-positive solid tumors, including those resistant to standard HER2-targeted therapies (86).

An investigation explored the expression of carcinoembryonic antigen (CEA) and HER2 in BCa and evaluated the potential of a BsAb termed BAb targeting both antigens for improved tumor uptake and residence time. Immunohistochemistry was initially performed on primary breast tumors, revealing that 65% of cases were positive for CEA, 19% for HER2, and 12% expressed both antigens. A BAb targeting CEA and HER2 was then developed and characterized. In the context of a double-positive tumor model (SKOv3-CEA-1B9), it was observed that the BAb displayed comparable internalization patterns to the 35A7 F(ab’)2-PDM despite its dual specificity. Interestingly, the BAb exhibited a notably higher degree of uptake in comparison to the FWP51 F(ab’)2-PDM, with the disparity becoming more pronounced 72 hours post-injection (7.3 ± 2.1% as opposed to 1.4 ± 0.5% of the injected dose per gram of tissue). This investigation postulates that the concurrent targeting of two distinct TAAs, namely, CEA and HER2, on the same cellular entity via a Bispecific Antibody (BsAb) can potentially augment tumor localization when contrasted with single-specificity antibodies. Such an approach bears promise for enhancing the effectiveness of antibody-based therapeutic interventions in the context of BCa (87).

The potential of a mAb, DF3, and its BsAb DF3xH22 in mediating phagocytosis and cytolysis of MUC-1-expressing BCa cells was examined by monocyte-derived macrophages (114). MUC-1 is frequently expressed in adenocarcinomas, including 80% of BCas, while HER2 is overexpressed in approximately 30% (88, 115). The expression of MUC-1 and HER2 exhibits partial overlap but lacks coordination. Consequently, concurrently targeting both antigens with antibodies may broaden the scope of patients eligible for immunotherapeutic interventions. The study outcomes revealed that Monoclonal Antibody (MAb) DF3 and Bispecific Antibody (BsAb) DF3xH22 both facilitated Antibody-Dependent Cellular Phagocytosis (ADCP). MAb DF3 exhibited a more pronounced ADCP activity than BsAb DF3xH22, while neither antibody induced ADCC. Interestingly, the inclusion of interferon-gamma (IFN-γ) in monocyte-derived macrophage cultures led to a suppression of ADCP in contrast to the presence of GM-CSF alone. Immunohistochemical analysis of primary BCa tissues depicted a partially overlapping yet non-coordinated expression pattern of MUC-1 and HER2 across the 67 cases examined. Based on these findings, the authors recommend simultaneously targeting MUC-1 and HER2 in BCa due to their partially overlapping expression profiles. MAb DF3 and BsAb DF3xH22 effectively facilitate target cells expressing MUC-1 phagocytosis. Further investigations are required to ascertain whether this antibody-triggered phagocytosis leads to sustained and specific T-cell activation against MUC-1 (114).

The study aimed to improve the therapeutic efficacy of PEGylated liposomal doxorubicin (PLD) in patients with HER2-overexpressing BCa. PLD is often ineffective in these patients due to their intrinsic low sensitivity to doxorubicin (89). The researchers developed a humanized BsAb (BsAb; mPEG × HER2) targeting methoxy-polyethylene glycol (mPEG) and HER2. The primary objective of this study was to augment the specificity, internalization, and anticancer efficacy of PEGylated Liposomal Doxorubicin (PLD) in cancer cells characterized by HER2 overexpression. Through a one-step formulation process, the investigators integrated PLD with mPEG × HER2 to create liposomes specifically targeted to HER2. These liposomes exhibited stability under conditions of both 4°C in phosphate-buffered saline (PBS) and 37°C in the presence of serum. The inclusion of αHER2/PLD, denoting the targeted liposomes, facilitated receptor-mediated endocytosis and increased doxorubicin accumulation within HER2-amplified BCa cells (MCF7/HER2). The cytotoxicity of αHER2/PLD was notably elevated, demonstrating more than a 200-fold enhancement in MCF7/HER2 cells and a 28-fold increase in drug-resistant MDA-MB-361 cells characterized by a deletion in the *TOP2A* gene. In an *in vivo* mouse model featuring tumor-bearing mice, αHER2/PLD exhibited a specific accumulation of doxorubicin in the nuclei of cancer cells. Compared to untargeted PLD, this targeted approach resulted in significantly enhanced antitumor efficacy against both MCF7/HER2 and MDA-MB-361 tumors. Importantly, αHER2/PLD demonstrated cardiotoxicity similar to that of PLD in both human cardiomyocytes and murine models. The findings of this investigation propose that the one-step formulation of PLD employing mPEG × HER2 represents a straightforward method to heighten tumor specificity, increase drug internalization, and enhance the anticancer activity of PLD against BCa cases characterized by HER2 overexpression and resistance to doxorubicin. This approach can potentially ameliorate the limited sensitivity of HER2-positive BCa to PLD and subsequently improve treatment outcomes (89).

Researchers have developed an anti-EGFR/HER2BsAb called TC-BsAb to address the limitations of anti-HER2 therapies. TC-BsAb is engineered by combining trastuzumab with cetuximab, an anti-EGFR chimeric antibody (90). The administration of TC-BsAb results in the internalization of both EGFR and HER2 receptors, in contrast to trastuzumab and cetuximab when used individually or in combination, which fail to induce the internalization of HER2. This observation suggests that TC-BsAb operates through a distinct and unique mechanism compared to the individual antibodies. In both *in vitro* and *in vivo* experiments, TC-BsAb displayed a notably higher efficacy in inhibiting the proliferation of BCa cell lines when compared to trastuzumab, cetuximab, or the combination of trastuzumab and cetuximab. These findings indicate the potential of TC-BsAb as a promising therapeutic approach for BCa treatment. It is essential to emphasize that further investigations and clinical trials are imperative to substantiate the effectiveness and safety of TC-BsAb in BCa patients. Nonetheless, developing BsAbs, such as TC-BsAb, opens new avenues for enhancing treatment outcomes in BCa cases characterized by HER2 overexpression and addresses the limited response to current therapeutic modalities (90).

The study’s findings reveal that EGFR and vascular endothelial growth factor receptor 2 (VEGFR2) are frequently overexpressed in TNBC and cooperate in an autocrine and paracrine manner to facilitate tumor growth and angiogenesis (116). While mAbs targeting EGFR (e.g., cetuximab) and VEGFR2 (e.g., ramucirumab) have received FDA approval for various cancer types, they are not currently sanctioned for treating BCas. In TNBC, VEGF-A secreted by cancer cells exerts paracrine effects by promoting angiogenesis in endothelial cells and simultaneously stimulates cancer cell growth via autocrine signaling (117). To interrupt this autocrine/paracrine loop and concurrently target the EGFR-mediated tumor growth signaling and the VEGFR2-mediated angiogenic pathway, the investigators devised a BsAb, specifically an anti-EGFR/VEGFR2 BsAb. Utilizing a glycine linker, this BsAb was created by combining the IgG backbone of cetuximab with the scFv of ramucirumab. The physicochemical characterization of the anti-EGFR/VEGFR2 BsAb demonstrated its ability to bind to both EGFR and VEGFR2 with a binding affinity similar to that of the parental antibodies. The BsAb exhibited anti-tumor activity *in vitro* and *in vivo* using TNBC models. Mechanistically, the anti-EGFR/VEGFR2 BsAb directly inhibited EGFR and VEGFR2 in TNBC cells, thus disrupting the autocrine mechanism in a TNBC xenograft mouse model. Additionally, it blocked ligand-induced activation of VEGFR2 and thwarted the paracrine pathway mediated by VEGF, which was secreted from TNBC cells and impacted endothelial cells. These innovative findings underscore the multifaceted mechanisms by which the anti-EGFR/VEGFR2 BsAb impedes tumor growth. Consequently, further investigation is warranted to explore its potential as a targeted antibody therapeutic for TNBC treatment (91).

Resistance to therapies targeting VEGF-A and VEGF-R2 is observed in many tumor models (118). In light of this, it has been found that blocking both the DLL4-Notch and VEGF signaling pathways simultaneously can have a synergistic effect in inhibiting tumor blood vessel density and function, ultimately reducing tumor growth (119). A bispecific mAb named HB-32 has been successfully developed, targeting human DLL4 and VEGF. HB-32 has demonstrated high binding affinity to VEGF and DLL4 (120). *In vitro* experiments have shown that HB-32 effectively inhibits the proliferation, migration, and tube formation of human umbilical vein endothelial cells (HUVEC), which are involved in angiogenesis. Furthermore, *in vivo* xenograft studies using BCa cells (MDA-MB-231) have been conducted. These studies have demonstrated that HB-32 inhibits the proliferation of BCa cells and induces tumor cell apoptosis more effectively than treatment with an anti-VEGF antibody or an anti-DLL4 antibody alone. These findings suggest that the BsAb HB-32 holds promise as a potential treatment for BCa. By targeting DLL4 and VEGF, HB-32 exhibits enhanced anti-tumor effects compared to single-targeting antibodies. However, further research and clinical trials are necessary to fully evaluate the efficacy and safety of HB-32 as a therapeutic option for BCa (92).

The prolactin receptor (PRLR) plays a significant role in certain breast and prostate cancers, making it an attractive target for cancer treatment (121). However, previous attempts to block PRLR have shown limited effectiveness despite being safe (122). In another investigation, the trafficking and internalization of cell surface proteins targeted by antibody-drug conjugates (ADCs) were compared (93). Specifically, the trafficking of HER2, the ado-trastuzumab emtansine (T-DM1) ADC’s target, was compared to that of PRLR, another potential target in BCa (113). The researchers found that PRLR undergoes rapid and constitutive internalization and efficiently traffics to lysosomes, where it is degraded. They also discovered that the cytoplasmic domain of PRLR plays a crucial role in promoting its internalization and degradation. Interestingly, when the PRLR cytoplasmic domain was transferred to HER2, it enhanced the degradation of HER2. Based on these findings, the study showed that low levels of cell surface PRLR (approximately 30,000 receptors per cell) were sufficient for effective killing by a PRLR ADC. In contrast, higher levels of cell surface HER2 (approximately 106 receptors per cell) were required for cell killing by a HER2 ADC. Moreover, the investigators demonstrated that the non-covalent linkage of HER2 to PRLR at the cellular membrane, achieved by using a BsAb capable of binding to both receptors, led to a significant enhancement in HER2 degradation and the cytotoxic effect of a non-competing HER2 ADC. In BCa cells where HER2 and PRLR were coexpressed, a HER2xPRLR bispecific ADC exhibited superior cell-killing activity compared to a HER2-specific ADC. These results underscore the pivotal role of intracellular trafficking in determining the efficacy of ADC targets. They suggest that tethering an ADC target to a rapidly internalizing protein, such as PRLR, can heighten the internalization process and the cell-killing potential of ADCs. This novel approach promises to enhance the therapeutic effectiveness of ADC-based treatments in BCa and potentially other cancer types (93).

Another study developed a novel BsAbs, PRLR-DbsAb, which can simultaneously target PRLR and CD3 on the surface of T cell (94). By engaging the immune system, this antibody enhances the body’s natural defenses against cancer cells expressing PRLR. PRLR-DbsAb successfully activated T cells and stimulated the release of antitumor cytokines that help kill BCa cells. Animal studies using mouse models further demonstrated the potential of PRLR-DbsAb as a therapeutic option, showing significant inhibition of tumor growth and increased survival compared to traditional mAb treatment (94). These findings highlight the promise of immunotherapy, explicitly targeting PRLR, as a potential avenue for effective cancer treatment. However, further research and clinical trials are necessary to fully explore the therapeutic potential of PRLR-DbsAb and its impact on human patients with PRLR-expressing cancers.

MDX-210 is a BsAb designed to target HER2 and Fc gamma receptor I (FcγRI) (123). Notably, HER2 is overexpressed in approximately 30% of BCa patients, and FcγRI is present on the surface of specific immune cells. In an examination of the capacity of MDX-210, its partially humanized counterpart MDX-H210, and the parental mAb 520C9 (anti-HER2/neu) to induce phagocytosis and cytolysis of BCa cells by human monocyte-derived macrophages (MDMs), the results revealed that both MDX-210 (via FcγRI) and 520C9 (via FcγRII) facilitated similar levels of antibody-dependent cellular phagocytosis (ADCP) and ADCC. MDX-H210, the partially humanized variant of MDX-210, exhibited equivalent ADCP activity compared to MDX-210. Confocal microscopy corroborated that the dual-labeled cells represented bona fide phagocytosis. It was noted that ADCP and ADCC were more pronounced when MDMs were pre-incubated with granulocyte-macrophage colony-stimulating factor (GM-CSF) compared to macrophage colony-stimulating factor (M-CSF). The study established that MDX-210 was as effective as the parental antibody 520C9 in stimulating phagocytosis and cytolysis by MDMs *in vitro*. Furthermore, MDX-210 and MDX-H210 demonstrated similar levels of ADCP activity (95). These findings support the ongoing clinical investigations of MDX-210 and its partially humanized derivative as potential treatments.

TNBC poses a significant medical challenge due to its unfavorable prognosis and limited therapeutic options (124). Mesothelin, a membrane protein with limited normal tissue expression but frequently elevated levels in a substantial portion of TNBC cases, has garnered attention as a promising target for therapy (125). Overexpression of mesothelin in breast tumors is linked to reduced disease-free survival and an increased incidence of distant metastases (125). To explore an immunotherapeutic approach based on BsAb, which simultaneously targets mesothelin and engages CD16, a Fab-like bispecific format named MesobsFab was employed (96). *In vitro* experiments utilized two TNBC cell lines characterized by varying surface mesothelin expression levels and distinct epithelial/mesenchymal phenotypes. The results indicated that MesobsFab effectively facilitated the recruitment and infiltration of natural killer (NK) cells into tumor spheroids, elicited dose-dependent cell-mediated cytotoxicity against mesothelin-positive tumor cells, triggered cytokine secretion, and mitigated cell invasiveness. MesobsFab also induced cytotoxicity in quiescent human PBMC, primarily through its NK cell-mediated ADCC activity. In *in vivo* experiments, the therapeutic efficacy of MesobsFab correlated with the density of mesothelin on the target cells (96). These findings underscore the significance of mesothelin as a pertinent therapeutic target, particularly in the subset of TNBC cases characterized by mesothelin overexpression, which is associated with dismal overall and disease-free survival rates. Moreover, this study highlights the potential of MesobsFab as an antibody-based immunotherapeutic agent for TNBC, demonstrating its capacity to augment immune-mediated anti-tumor responses and curb tumor invasiveness.

Trastuzumab is a well-established treatment for HER2-positive metastatic BCas, but various factors often limit its efficacy (126). A BsAb called HER2bsFab with a moderate affinity for HER2 and a unique, high affinity for FcγRIII was designed for BCa treatment (97). *In vitro* characterization of HER2bsFab showed that its major mechanism of action is ADCC, as no remar HER2-driven effect was detected. HER2bsFab demonstrated potent ADCC activity at very low concentrations against HER2-high, HER2-low, and trastuzumab-refractory cell lines. *In vivo*, studies have shown that HER2bsFab effectively inhibited the growth of HER2-high tumors by recruiting resident effector cells expressing mouse FcγRIII and IV. Importantly, HER2bsFab showed superior inhibition of HER2-low tumor growth compared to trastuzumab. Additionally, engagement of FcγRIIIA by HER2bsFab was not dependent on the V/F158 polymorphism and induced more robust activation of NK cells upon recognition of target cells. Overall, HER2bsFab exhibited potent anti-tumor activity against HER2-low tumors while overcoming most of the Fc-related limitations of trastuzumab. By combining its specificity and affinity for both HER2 and FcγRIIIA, HER2bsFab has the potential to expand the eligibility of patients for BCa immunotherapy, offering a promising approach to overcome the limitations of current treatments. However, further research and clinical trials are necessary to validate the effectiveness and safety of HER2bsFab in BCa patients (97).

In a study, a trivalent BsAb targeting HER2 and CD16 was developed. This BsAb was designed to physically cross-link immune cells, specifically NK cells, to tumor cells, promoting cellular cytotoxic mechanisms and enhancing anti-tumor immune responses (98). The BsAb was engineered with bivalent arms that specifically bind to the extracellular domain of ErbB2, a receptor overexpressed in certain tumors, and monovalent Fab fragments that redirect NK cells. The functionality of the BsAb was confirmed through its ability to bind to both SKBR3 tumor cells and NK cells in a bispecific manner. One advantage of this trivalent BsAb is its molecular size, which falls between that of a diabody (smaller antibody fragment) and a whole antibody. This size is expected to provide benefits such as better tissue penetration due to the smaller size and slower clearance from circulation compared to complete antibodies. Collectively, this novel trivalent BsAb holds promise as a therapeutic agent for targeting ErbB2-positive tumors and activating NK cells to enhance anti-tumor immune responses. Further improvements and evaluations are warranted to optimize its efficacy and potential clinical applications (98).

### 4.2 Clinical studies

This section discussed the most important clinical studies in BCa patients treated with various types of BsAbs (**Table 2**). As mentioned earlier, MDX-H210 is a BsAb composed of antigen-binding fragments (F(ab’) fragments) of mAb H22, which binds to FcγRI, and mAb 520C9, which targets HER2. This BsAb has demonstrated tumor cell lysis *in vitro* and mouse models expressing human FcγRI. FcγRI is a potent signaling molecule that is expressed on monocytes, macrophages, immature DCs, and granulocyte colony-stimulating factor (G-CSF)-stimulated polymorphonuclear cells (PMN) (132, 133). An investigation focused on using myeloid cells, specifically FcγRI (CD64)-expressing monocytes/macrophages and G-CSF-primed neutrophils, as effector cells for tumor cell cytotoxicity mediated by specific immunoglobulin receptors (127). *In vitro* experiments demonstrated that MDX-210 effectively induced lysis of HER2 overexpressing BCa cell lines. Further assays revealed that FcγRI-positive neutrophils were a significant population of effector cells during G-CSF therapy. Building on these preclinical findings and a previous study at Dartmouth, a phase I clinical trial was conducted in BCa patients to test the combination of G-CSF and MDX-210. In this study, patients receiving G-CSF were treated with escalating single doses of MDX-210. The therapy was generally well tolerated, although some patients experienced fever and short periods of chills, which correlated with elevated plasma levels of IL-6 and TNF-α. Following MDX-210 administration, a temporary decrease in total white blood count and absolute neutrophil count (ANC) was observed. However, *in vitro* experiments showed that isolated neutrophils from patients undergoing G-CSF treatment displayed high cytotoxicity in the presence of MDX-210. These findings suggest a potential role for G-CSF and BsAb in immunotherapy for BCa. By harnessing the cytotoxic capabilities of FcγRI-expressing myeloid cells, specifically neutrophils, in combination with HER2 targeting, this approach holds promise for enhancing anti-tumor immune responses. Further research and clinical trials are needed to assess the efficacy and safety of this combination therapy (127).

**Table 2:** The most important clinical studies using BsAbs in BCa.

BsAbs	BsAbs Targets	Details of study	Outcomes	Ref/NCT
**Combination of G-CSF and MDX-210**	HER2 and FcγRI	*In vitro* *In vivo* Phase I clinical trial	•Effectively induced lysis of HER2 overexpressing BCa cell lines •The therapy was generally well tolerated although some patients experienced fever and short periods of chills which correlated with elevated plasma levels of IL-6 and TNF-α •A decrease in total WBC count and ANC •Isolated neutrophils from patients undergoing G-CSF treatment displayed high cytotoxicity in the presence of MDX-210	(127)
**Combination of G-CSF and MDX-210**	HER2 and FcγRI	Phase I clinical trial	•Common side effects included fevers in 19 patients diarrhea in 7 patients and allergic reactions in 3 patients which did not necessitate discontinuation of therapy •The beta-elimination half-life of MDX-H210 ranged from 4 to 8 hours at doses up to 20 mg/m2 •Release of cytokines IL-6 G-CSF and TNF-α •Increasing human anti-BsAb after the third infusion •No objective clinical responses	(128)
**KN026**	HER2 (domain II and IV) From heavy chains of pertuzumab and trastuzumab^27^ with a common light chain	KN026-CHN-001 Phase I first-in-human multicenter open-label single agent dose-escalation and dose-expansion study	•Increased ORR and median PFS in patients with co-amplification of HER2/CDK12	(129) NCT03619681
**HER2 BATs**	HER2 and CD3 Two cross-linked mAbs	Phase II clinical trial	•Increased Th1 cytokines Th2 cytokines and chemokines were observed after HER2 BATs infusions •Enhanced adaptive and innate antitumor responses Immune consolidation with HER2 BATs after chemotherapy increased the proportion of patients who remain stable at four months and improves the median OS for both HER2-HR^+^ and TNBC patient groups	(130) NCT01022138
**HER2Bi armed anti-CD3–activated T cells in combination with low-dose IL-2 and GM-CSF**	HER2 and CD3 BsAb sources: Trastuzumab heteroconjugated to OKT3	Phase I clinical trial	•Increasing OS •Increasing IFN-γ and Th1 cytokines in the patient’s blood indicating enhanced immune responses. These infusions induced •Inducing antigen-specific T cell and antibody responses against HER2 CEA and EGFR	(131) NCT00027807

In another phase I clinical trial, the primary objective was to investigate the utilization of the humanized BsAb MDX-H210 in conjunction with G-CSF in patients afflicted with metastatic BCa (MBCa) displaying overexpression of HER2 (128). The study encompassed several key aims, which encompassed establishing the maximum tolerated dose (MTD) of MDX-H210 when administered alongside G-CSF, characterizing the pharmacokinetic profile of MDX-H210 when used in combination with G-CSF, assessing the treatment’s toxicity, biological effects, and its potential therapeutic efficacy. The treatment regimen involved administering MDX-H210 weekly for three doses, followed by a 2-week hiatus and an additional three weekly doses. A total of 23 patients were recruited for this trial, and the doses of MDX-H210 were incrementally escalated from 1 mg/m2 to 40 mg/m2, with the MTD not being reached. The adverse effects linked to the combination of MDX-H210 and G-CSF were relatively manageable, and no dose-limiting toxicity was observed. Common side effects included fever in 19 patients, diarrhea in 7 patients, and allergic reactions in 3 patients, none of which necessitated the discontinuation of therapy. The beta-elimination half-life of MDX-H210 spanned from 4 to 8 hours at doses up to 20 mg/m2. A significant release of cytokines IL-6, G-CSF, and TNF-α was observed after administering the BsAb. Flow cytometric analysis indicated the binding of MDX-H210 correlated with the disappearance of circulating monocytes within 1 hour of infusion. The plasma of most patients showed significant levels of human anti-BsAb after the third infusion. However, this cohort of heavily pre-treated patients observed no objective clinical responses. Although the study did not demonstrate significant therapeutic efficacy regarding clinical responses, it provided valuable information regarding the toxicity profile, pharmacokinetics, and biological effects of MDX-H210 in combination with G-CSF. Further studies may be warranted to explore alternative treatment strategies or combinations to improve outcomes for patients with MBCa overexpressing HER2 (128).

A phase I clinical trial was conducted to assess various aspects of KN026, a novel BsAb with the unique property of targeting two distinct HER2 epitopes, akin to the mechanisms of action of trastuzumab and pertuzumab. This study primarily focused on examining its safety profile, pharmacokinetics, initial therapeutic effectiveness, and the potential of certain biomarkers to predict its activity. The clinical trial was carried out on a group of female patients afflicted with MBCa characterized by HER2 overexpression, who had previously exhibited disease progression while undergoing anti-HER2 therapies (129). KN026 was administered as a standalone treatment, with varying dosages of 5 mg/kg once weekly, 10 mg/kg once weekly, 20 mg/kg once every two weeks, or 30 mg/kg once every three weeks. The trial adhered to a dose escalation procedure based on the “3 + 3” rule, followed by a subsequent expansion of dose levels. A total of 63 patients were recruited for this study. The adverse events associated with KN026 treatment, which were attributed to the intervention itself, included symptoms such as fever (referred to as pyrexia), diarrhea, elevated levels of aspartate aminotransferase, and increased alanine aminotransferase levels in the blood. Notably, severe (Grade III) treatment-related adverse events were observed in only four patients, indicating that the safety profile of KN026 was generally manageable. An analysis of the relationship between the exposure to the drug and the observed response supported the identification of recommended doses for phase II trials, which were determined to be either 20 mg/kg administered once every two weeks or 30 mg/kg once every three weeks. In a subset of 57 patients, these doses yielded objective response rates (ORR) of 28.1% and a median progression-free survival (PFS) of 6.8 months, with a 95% confidence interval spanning from 4.2 to 8.3 months. Furthermore, translational research conducted on a subgroup of 20 patients who exhibited HER2 gene amplification provided valuable insights. This research confirmed that the concurrent amplification of the CDK12 gene, which is involved in the regulation of the cell cycle, in conjunction with HER2, served as a promising biomarker for predicting a more favorable response to KN026 treatment. Patients demonstrating co-amplification of HER2 and CDK12 achieved an ORR of 50% and a median PFS of 8.2 months, in stark contrast to patients who lacked this co-amplification, where the ORR was 0% and the median PFS was limited to 2.7 months. This noteworthy discovery underscores the potential utility of HER2/CDK12 co-amplification as a predictive biomarker, offering a means of identifying patients who are more likely to experience positive therapeutic outcomes when treated with KN026 (134). Therefore, KN026, a BsAb targeting HER2, exhibited a favorable safety profile and achieved therapeutic efficacy that was comparable to the combination of trastuzumab and pertuzumab, even in patients who had undergone extensive prior treatment. The presence of co-amplification of HER2 and CDK12 may serve as an important predictive biomarker for identifying patients with a greater likelihood of responding positively to KN026 therapy (129).

In a phase II clinical trial, the study investigated the effectiveness of anti-CD3 × anti-HER2 BsAb equipped activated T cells, referred to as HER2 BATs, in patients with metastatic BCa who lacked HER2 overexpression, including those with HER2-estrogen and/or progesterone receptor-positive (HR^+^) tumors as well as those with TNBC (130). The primary objective of the trial was to extend the typical duration of disease progression following the ineffectiveness of first-line therapy, with secondary objectives focusing on enhancing overall survival and stimulating immune responses. The trial enrolled 24 patients with HER2-HR^+^ BCa and 8 patients with TNBC. The HER2-HR^+^ patients had an average of 3.75 prior lines of chemotherapy, while the TNBC patients had an average of 2.4 prior lines of chemotherapy. Patients received HER2 BAT infusions on a weekly basis for three weeks, with an additional booster dose administered after 12 weeks. Among the 32 patients who could be evaluated, eight maintained stable disease four months after the first infusion. Notably, no dose-limiting toxicities were observed during the course of treatment. Tumor markers declined in 13 out of 23 patients with available tumor marker data. The median OS for the entire patient cohort was 13.1 months (with a 95% confidence interval ranging from 8.6 to 17.4 months). Specifically, HER2-HR+ patients exhibited a median OS of 15.2 months (95% CI: 8.6 to 19.8 months), while TNBC patients had a median OS of 12.3 months (95% CI: 2.1 to 17.8 months). Within patients who had either chemotherapy-sensitive or chemotherapy-resistant disease following prior chemotherapy, the median OS was 14.6 months (95% CI: 9.6 to 21.8 months) and 8.6 months (95% CI: 3.3 to 17.3 months), respectively. Moreover, the study observed significant increases in interferon-γ immunospots, Th1 cytokines, Th2 cytokines, and chemokines following the infusions of HER2 BATs, indicating an enhancement in both adaptive and innate antitumor responses (130). These findings suggest that employing HER2 BATs for immune consolidation after chemotherapy increases the proportion of patients who maintain stable disease at the four-month mark and improves the median OS for both the HER2-HR+ and TNBC patient groups. The study also underscores the enhancement of adaptive and innate antitumor responses. Future investigations exploring the combination of HER2 BATs with checkpoint inhibitors or other immunomodulators may offer further potential for improving clinical outcomes.

In a phase I clinical trial, researchers studied the effects of infusing HER2 BATs (HER2-targeted adoptive T cells) in 23 women with HER2 0–3+ MBCa. The median OS for these patients was 37 months. Specifically, the patients with HER2 3+ tumors had a median OS of 57 months, while those with HER2-negative (0–2+) tumors had a median OS of 27 months. This suggests that HER2 BAT infusions may positively impact survival, especially in patients with HER2 3+ tumors. Additionally, HER2 BAT infusions significantly increased IFN-γ ELISpots responses and Th1 cytokines in the patient’s blood, indicating enhanced immune responses. These infusions induced antigen-specific T-cell and antibody responses against HER2, CEA, and EGFR. These immune responses could also be transferred to other patients using immune ATC (adoptive T cell therapy) expanded from individuals who had received HER2 BAT infusions. This study suggests that HER2 BAT infusions may improve survival in women with HER2-positive metastatic BCa by enhancing immune responses against cancer-related antigens (131).

## 5 Challenges and opportunities

**Figure 3:**
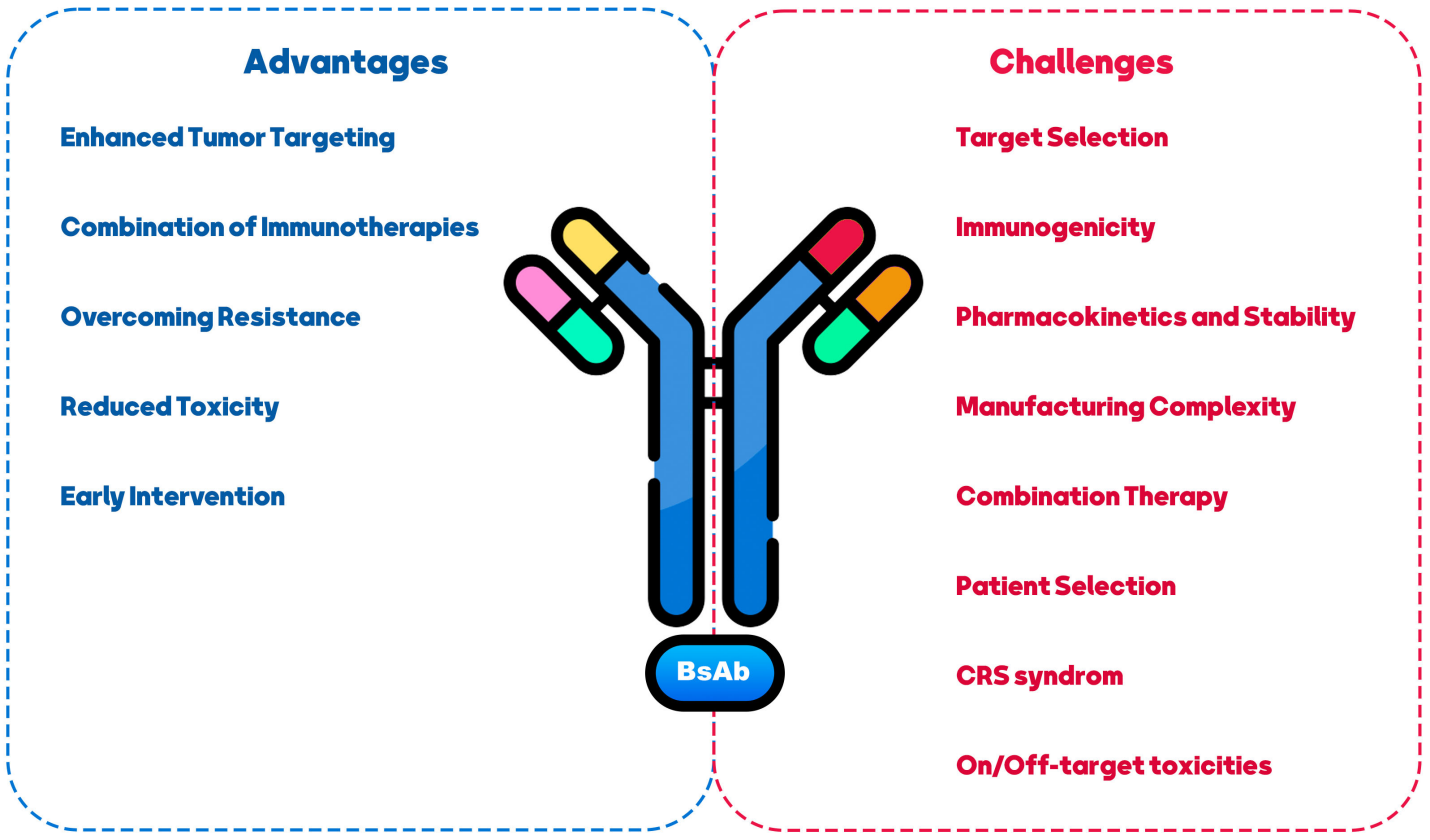
Challenges and opportunities in using BiAs as a therapeutic approach for breast cancer treatment.

The use of BsAb in BCa treatment presents both challenges and opportunities. This section summarized the most significant challenges and achievements of treating BCa with BsAbs (**Figure 3**).

### 5.1 Challenges

One of the primary challenges in developing BsAbs for various disease types revolves around the potential occurrence of CRS and autoimmune toxicities when administering BsAbs targeting CD3 and co-stimulation receptors (135, 136). These challenges are categorized into two areas of concern: “on-target/on-tumor” and “on-target/off-tumor” toxicities (137). The “on-target/on-tumor” toxicity typically arises from the engagement of the tumor antigen with the T-cell receptor (TCR), leading to cytokine release. In such cases, strategies like steroid administration, drug dosage, and distribution adjustments can often effectively manage this toxicity. Conversely, CRS is often driven by transient increases in pro-inflammatory cytokines such as TNFα, IL-6, IFNγ, and CCL2. Conversely, addressing the “on-target/off-tumor” toxicity of CD3-based BsAbs on normal tissue poses a greater challenge. This challenge is influenced by factors like the distribution of the target, the level of its expression on normal tissue, and the cellular localization of the target (138).

Animal models that predict BsAb-driven toxicities have proven unreliable in forecasting toxicities in human patients. The severity of CRS may correlate with the expression level of the target antigen in normal tissues. Ongoing clinical trials have observed histological changes such as lymphocytic infiltrates, acute inflammatory responses, and single-cell necrosis following the infusion of CD3 platform effector-based BsAbs (138). Ultimately, the outcomes of these clinical trials will provide valuable insights into the choice of effector cells to be targeted *in vivo* and the optimal dosing schedule, whether it involves a single or multiple administrations.

Identifying appropriate antigen targets in BCa is also challenging (138). BCa is a heterogeneous disease with various subtypes, and the target choice should consider each subtype’s specific characteristics (139). Selecting targets highly expressed on cancer cells and having functional relevance in promoting tumor growth or survival is essential (140). Moreover, generating BsAbs can sometimes lead to immunogenicity concerns (141). Introducing non-human components or creating novel antibody formats can potentially trigger immune responses in patients (142). Careful design and engineering strategies are necessary to minimize immunogenicity risks and ensure the safety and efficacy of BsAbs. BsAbs may exhibit altered pharmacokinetic profiles compared to traditional mAbs, such as rapid clearance, reduced half-life, or increased susceptibility to degradation, which can impact their efficacy (143, 144). Addressing these challenges through appropriate modifications, such as antibody half-life extension technologies, can enhance their stability and therapeutic potential (145). The production of BsAbs can be more complex than mAbs due to their dual-targeting nature (146). Manufacturing may require advanced techniques, including antibody engineering, purification, and quality control (147). Developing scalable and cost-effective manufacturing strategies is essential to facilitate BsAb therapies’ widespread availability and affordability (148). BCa treatment often involves a multi-modal approach, combining different therapeutic agents (149). BsAbs offer opportunities for combination therapy by targeting multiple pathways simultaneously (150, 151). However, the selection and timing of combination therapies should be carefully evaluated to maximize synergistic effects and minimize potential toxicities (58). Personalized medicine approaches should be considered when using BsAbs in BCa treatment (152). Identifying patients most likely to benefit from BsAb therapy based on biomarkers, genetic profiling, or other predictive factors can optimize treatment outcomes and minimize unnecessary side effects (153, 154).

### 5.2 Opportunities

Despite the mentioned challenges, BsAbs in BCa treatment presents several opportunities. BsAbs can improve tumor targeting by simultaneously binding to cancer cells and immune cells, redirecting the immune system to attack the tumor (37). This approach can overcome the limitations of tumor heterogeneity and increase the precision and effectiveness of treatment (155). BsAbs can be combined with other immunotherapeutic agents, such as ICIs or cancer vaccines, to enhance anti-tumor immune responses (100, 155, 156). Synergistic effects may be achieved by activating multiple immune pathways and overcoming immunosuppressive mechanisms within the TME. Resistance to targeted therapies is a significant challenge in BCa treatment (157). BsAbs can potentially target multiple signaling pathways simultaneously, addressing resistance mechanisms and improving treatment responses in resistant or refractory BCa cases (7, 158). BsAbs have the advantage of explicitly targeting cancer cells and sparing normal cells, potentially reducing off-target toxicities associated with non-specific treatments (80). This selective targeting may improve patient safety profiles and tolerability (159). BsAbscan can be utilized in earlier stages of BCa, including minimal residual disease or adjuvant settings, to prevent relapse and improve long-term outcomes (106, 160). The ability to engage the immune system and eradicate minimal residual disease may lead to more persistent anti-tumor immune responses, improving survival.

## 6 Concluding remarks

In conclusion, BsAbs offer exciting opportunities for BCa treatment by leveraging their unique targeting capabilities and the potential to engage the immune system. Addressing CRS, target selection, immunogenicity, manufacturing complexity, and patient selection will be critical to realizing the complete therapeutic.

## Author contributions

H-RL: Writing – original draft. MC: Writing – original draft. S-YY: Writing – original draft. J-XC: Writing – original draft, Writing – review & editing. K-TJ: Writing – original draft, Writing – review & editing.
